# John Pridmore Raban

**Published:** 1949-10

**Authors:** 


### JOHN PRIDMORE RABAN

*T.D.*, M.R.C.S. Eng., L.R.C.P. Lond., D.M.R.E.

DR. JOHN PRIDMORE RABAN died in Bristol after a very short illness on October 2nd at the early age of forty-four. Born at Harpenden, Herts., he received his medical education at the Middlesex Hospital, and qualified in 1929. After holding several resident appointments, he decided to make radiotherapy his career, and was appointed to what has since become the Meyerstein Institute of Radiotherapy. He acquired at this time the diploma of D.M.R.E., and continued to gain experience in this speciality until the outbreak of war.

In his student days John Raban was a keen member of the University of London O.T.C., and on qualifying he took a commission in the R.A.M.C. (T.A.). The training so gained stood him in good stead during the war years. After a period as radiologist to a General Hospital in India, he was given command of one of the first two units of its kind—a Forward Malarial Treatment Unit. This unit was sent into the Burmese jungle where its operational success was outstanding, and recognition of this was given by John Raban's Mention in Dispatches. His final war appointment was to the command, as full Colonel, of a General Hospital in Ceylon.

In 1945 he returned to the Middlesex as first assistant in the Meyerstein Institute, and was subsequently appointed Director of Radiotherapy in the United Bristol Hospitals. It was about this time that his health began to fail, and there can be no greater testimony to his courage and tenacity of purpose than the success he made of this responsible task against increasing odds. He stuck to his job until a few days before his death.

Raban's judgment was respected by all. His was a strong character which enabled him to speak his mind in all circumstances, and was shown perhaps best in his great qualities of leadership. In both war and peace he earned the respect, loyalty and friendship of all those who served under him. We desire to express our sympathy with his widow.

